# Benefits of Integrated Social Care in the Management of Patients With Inborn Errors of Metabolism

**DOI:** 10.1002/jmd2.70023

**Published:** 2025-05-15

**Authors:** A. Selvanathan, S. Nazir, K. van Wyk, E. Simpson, V. Holmes, R. Hutton, F. White, B. C. Schwahn

**Affiliations:** ^1^ Manchester Centre for Genomic Medicine, St Mary's Hospital Manchester University NHS Foundation Trust, Health Innovation Manchester Manchester UK; ^2^ Division of Evolution & Genomic Sciences, School of Biological Sciences, Faculty of Biology, Medicine and Health University of Manchester Manchester UK

**Keywords:** child protection, dietary management, inborn errors of metabolism, medication compliance, social care, social work

## Abstract

The current cornerstone of the management of many small‐molecule inborn errors of metabolism (IEMs) is a combination of dietary therapy and medication, with evidence for improved clinical outcomes. However, the burden imposed on patients and families is substantial. Many families also have to manage this burden in conjunction with other medical, psychosocial, and financial stressors. Adherence to the recommended treatment can therefore be extremely challenging, sometimes leading to sustained derangement of biochemical parameters and/or clinical deterioration. The treating team needs to work with the family to determine an individualized optimal management strategy, with targets that can be pragmatically achieved. This paper focusses on the role of social care in assisting patients with a range of different small‐molecule IEMs, as well as their families and the medical team. We provide six case vignettes that illustrate how social care involvement, in addition to enhanced psychosocial support from the clinical team, resulted in improved outcomes. This included assisting with adjustment to a new diagnosis, exploring and addressing barriers to treatment adherence, and provision of ‘early help’ community supports. In some instances where this was not sufficient and risk of harm to the child was considered significant, social care involvement facilitated graded escalation from a “child in need” approach to formal child protection measures. We identified challenges in engaging social workers external to the metabolic team. This included a need for greater education about the medical condition and the risks associated with undertreatment, lack of protected time for metabolic case management, and a lack of preventative involvement of social workers during the initial hospitalization (impacting on patient rapport). We advocate for the integration of social care within the metabolic team as part of a more holistic model of care.

1

**Summary**

- Social care involvement can be a successful intervention to help identify and address barriers to treatment adherence, improving outcomes for patients with metabolic disorders and their families. Access to social workers integrated in the metabolic service can lower the referral threshold and reduce delays, making the intervention more effective.

## 2 Introduction

Small‐molecule inborn errors of metabolism (IEMs) are rare genetic disorders that affect the function of an enzyme, receptor or transporter, leading to failure of pathways involved in either the catabolism or storage of macromolecules (proteins, fats and carbohydrates). Clinical manifestations of these conditions are incredibly variable, though adverse effects on development, growth and neurological function are most common [1]. Most are also characterized by acute decompensations during catabolic periods (intercurrent illness, fasting, surgery) with subsequent long‐term sequelae. Unlike many other genetic diseases, small‐molecule IEMs are considered at least partially treatable. This includes intensive dietary management, medications in some cases, as well as proactive and reactive interventions during intercurrent illness (“unwell management plans”) [2]. More advanced therapeutic strategies such as mRNA and other genetic therapies are in clinical trial [3], but are mostly yet to be proven, and are extremely expensive.

While there is substantial evidence to support the use of dietary therapies and medications to manage small‐molecule IEMs, the burden imposed on families by this treatment is substantial. For instance, patients with disorders of amino acid metabolism are often limited to a very narrow diet (excluding all meat, fish, eggs, dairy products, legumes and even normal bread/pasta): other foods are often restricted to weighed allowances [4, 5]. In addition, disease‐specific amino acid supplements, as well as medications (such as glycine for isovaleric acidaemia), often need to be taken multiple times per day [6].

Unwell management plans are challenging in that they require further restriction of the potentially toxic macronutrient (such as protein or fat), while also providing increased calories (often 120%–130% of the caloric target when well). Timely initiation of the unwell management plan by parents/carers and ongoing daily communication with the medical team are important for avoiding hospitalization. In this way, the dietary interventions required are often far more stringent and intrusive than for other conditions requiring dietary restriction (such as Type 1 diabetes mellitus, or food allergy).

Unsurprisingly, this intensive management is extremely challenging for all families of children with small‐molecule IEMs. Many have to balance this management with other personal health issues, psychosocial stressors, and financial stressors [7, 8]. The difficulties in adherence can be seen at times through the sustained derangement of biochemical parameters (more prevalent in adolescence), upon which the treating team, child, and family usually work together to determine the optimal management strategy that can be pragmatically achieved [9].

This paper focuses on the role of social care in assisting patients with small‐molecule IEMs, as well as their families and the clinical team. We provide illustrative case vignettes detailing where and how social care involvement was beneficial and consider how the broader literature around non‐adherence can be applied in the metabolic context. Through this, we demonstrate the need for integrated social work involvement as part of any clinical metabolic service to facilitate a more holistic approach to management that can improve outcomes.

## 3 Methods

### 3.1 Metabolic Service Demographics

The study was conducted as a retrospective service review of patients with inborn errors of metabolism requiring dietary management, from a large tertiary metabolic service that provides metabolic support to a large part of the north of England. This includes a cohort of over 700 patients at any time who are dietetically treated and under long‐term follow‐up (until age 18 years). The majority of these patients have phenylketonuria: organic acidemias, maple syrup urine disease, hepatorenal tyrosinemia, genetic hyperlipidemias, and the glycogen storage diseases are the other common disorders.

### 3.2 Standard of Care for Disease Monitoring and Treatment

Most patients with IEMs are identified during the neonatal period. At the time of diagnosis, parents and other carers are routinely educated by the clinical team (comprised of specialized dietitians, nurses and doctors) about the metabolic disorder and its management, both verbally and with written information. There are specific dietary sessions on “introduction to solids,” usually conducted one‐on‐one with families, which assist families with counting macronutrients and food label reading if required: resources for low‐protein or low‐fat meal options, as well as high‐carbohydrate options when unwell, are also provided.

Pharmacodynamic biomarkers are often used for monitoring: the aim is to have the majority of biochemical results within the treatment target range (for instance in phenylketonuria, the target range for blood phenylalanine levels is 120‐360 μmol/L) [10]. Patients with disorders associated with acute decompensations (such as maple syrup urine disease) require further intensification of their dietary management when unwell, necessitating daily communication between the metabolic team and the family. Parents and older children are regularly trained how to select and prepare suitable food, how to take capillary blood samples for self‐monitoring, and in the use of emergency regimens. Results of self‐monitoring and dietary adjustments are directly communicated to carers on a frequent, often weekly basis to empower them to take charge of their own management. All of these activities are hospital‐based, originating from outside the home environment.

### 3.3 Pathway for Graded Escalation Prior to Social Care Involvement

**FIGURE 1:**
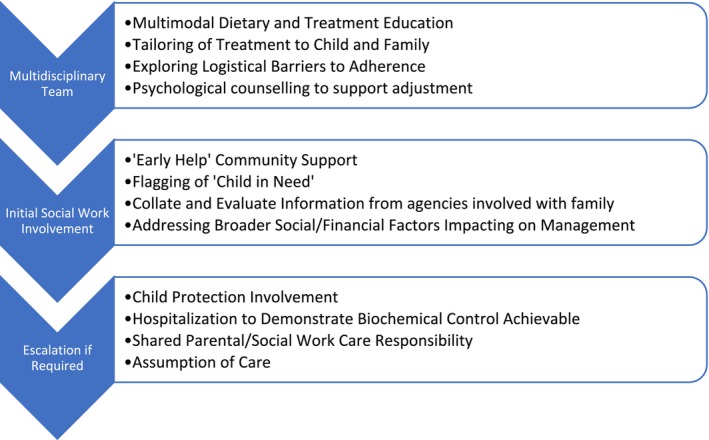
Algorithm for social care involvement in patients with inborn errors of metabolism. Note that in some centers a social worker is part of the metabolic multidisciplinary team, and can participate in the early management phase.

In the authors' experience, a significant minority of patients and their families have difficulties with adhering to the recommended management strategies at any given time: this becomes apparent through either biochemical monitoring or during unwell management periods. In such situations, additional interventions are organized without delay (see Figure 1): these include more frequent dietetic counseling with motivational interviewing and additional education often provided by nursing and dietetic staff. Recruitment of community support (school staff, the health visitor or community nurses for assistance with taking blood samples) is intensified, and recourse to charities for financial and emotional support is provided. A variety of modes of delivery are often trialed, including face‐to‐face discussion, video‐consulting as an alternative to in‐person visits (particularly for those living far away from the tertiary metabolic center) and frequent regular phone reviews. Based on identified needs, a referral is made to dedicated clinical psychologists with experience in the management of IEMs for counseling of parents or older children to support acceptance of the diagnosis and concordance with treatment recommendations.

If these measures are unsuccessful and there is a persistent failure to achieve treatment targets for longer than 6 months, a referral to social care is usually considered, although often effected with further delay. The aim of such a referral is to obtain engagement from social workers who can assess the wider socioeconomic situation of the family and may initiate support for a “child in need”, for example, with regular home visits by a support worker and by arranging MDT meetings of all involved professionals from education and health care providers and family members. Safeguarding measures may escalate to placing a child on a child protection register, or considering removal of the child from their carers.

### 3.4 Illustrative Cases

In our patient cohort, we identified 18 cases with formal involvement of social care over the last 10 years and evaluated referral criteria and factors contributing to a successful intervention. We focus particularly on six cases in whom social care referral resulted in sustained improvement in metabolic management. Given the nature of the topic, specific consent for publication was not obtained from the families. Cases are presented in an illustrative manner, and every effort was taken to ensure patient anonymity by choosing conditions that are relatively common in the field of inborn errors of metabolism, by avoiding mentioning specific biochemical values, and by removing all potentially identifying details.

## 4 Case Vignettes

### 4.1 Patient 1

Patient 1 was diagnosed on newborn screening with classical phenylketonuria. Phenylalanine levels were initially within the treatment target range (120–360 μmol/L) for the first 4 months of life but subsequently were elevated above the treatment range in over 60% of received samples. Adherence to a low‐protein diet and phenylalanine‐free supplements was self‐reported to be challenging at home, and dried blood spot samples for phenylalanine monitoring were also often either not sent or insufficient. There was irregular attendance at clinic appointments and difficulties contacting the family via phone and email to advise on management changes: attempts at further education and tailoring management to try and suit the family did not result in improved adherence. The child was able to attend mainstream school but was diagnosed with learning difficulties and inattention.

An inpatient admission was arranged which helped to reduce phenylalanine levels from > 1500 μmol/L to < 700 μmol/L before discharge against medical advice. Unfortunately, following this admission, the phenylalanine levels increased again, and hence a referral to social care was made to enable support of the family at home.

The social worker performed home visits, and facilitated case conferences regarding the difficulties Patient 1's family were having in following management recommendations. In addition, the social worker liaised with the housing officer to expedite outstanding repairs in the family home, reducing parental stress. They also acted as a point of contact for the family for ordering home‐delivered, phenylalanine‐free protein supplements. With social work team involvement, phenylalanine levels came down and interactions between the family and the healthcare team improved. For instance, at age six, the average phenylalanine levels across 1 month improved from 2.5 times the upper limit of the treatment target range, to within the treatment target range. During periods where the intensive regular social work support was withdrawn, the previous challenges re‐emerged.

### 4.2 Patients 2 and 3

Patient 2 was diagnosed with classical phenylketonuria on newborn screening. There was generally good control in the first year of life, with over 80% of phenylalanine levels in the treatment target range. Over the next 12 months, there were behavioral disturbances and difficulties in taking the phenylalanine‐free protein supplements, resulting in 80% of checked levels being above the treatment target range. Levels continued to be moderately elevated over the next few years (1.5–2.5 times the upper limit of the treatment range) despite attempts at behavioral intervention, and there was emerging clinical evidence of global developmental delay. Parental separation led to further behavioral deterioration and poor dietary adherence: dried blood spot monitoring became infrequent, and the child was not brought to most scheduled appointments.

Patient 3 is the younger sibling of Patient 2 and is also affected by classical phenylketonuria. Similar to the older child, in the first year of life most phenylalanine levels were within the treatment range, but results deteriorated from around 24 months of age onward. Despite frequent contact with the dietetic team where further educational and motivational strategies were provided, over 80% of levels were above the upper limit of the treatment range (120–360 μmol/L).

Given the known adverse developmental impact of sustained high phenylalanine levels [11], a referral to social services was made for both children. This resulted in mandated twice‐weekly dried bloodspot monitoring (with assistance from the social worker in posting the cards), psychological support for the family, and behavioral intervention for the children. Attempts at dietary modification were made (less normal‐protein snacks in the house, stronger routines in terms of mealtimes), and the parents sought attention for some of their own mental health challenges.

However, persistently elevated levels on the mandatory blood spot monitoring resulted in escalation to a child protection plan. The children were formally placed into foster care, with frequent parental visits permitted. Immediately after the change of care responsibilities, the siblings' levels dropped substantially: both patients had more than 60% of levels within the treatment target range. These improvements have been sustained for several years with this shared care arrangement.

### 4.3 Patient 4

Patient 4 initially presented with complex febrile seizures in early childhood and was also noted to have lens dislocation at this time requiring surgical intervention. The family came under our care when Patient 4 was a teenager. By this time, it was apparent that there were moderately severe learning difficulties as well as ongoing seizures.

Worsening seizures led to an acute hospital admission, and further investigations revealed a diagnosis of non‐pyridoxine responsive homocystinuria. Dietary treatment was started, including a low‐protein diet and amino acid formula supplementation. Over the next 12 months, there were difficulties with ensuring dietary compliance at home: likely due to the delayed diagnosis, the parents felt that the medical treatment offered was not necessary. This led to persistently elevated plasma total homocysteine concentrations (5–25 times the upper limit of normal) and multiple hospital admissions for refractory seizures. Attempted psychoeducational interventions by different members of the multidisciplinary team, including medical staff, nursing staff, and dietitians did not impact the frequency of these admissions, nor biochemical control.

Given the risk associated with severe hyperhomocysteinemia and the challenges encountered by the multidisciplinary clinical team, a referral to social care was made. Monthly safeguarding meetings were initially organized, which rapidly escalated to further child protection measures because of evidence of increased risk from ongoing high homocysteine levels (including seizure‐related hospital admissions). Increased supervision, home visits, and facilitating financial support led to greater engagement from the family, resulting in the patient's total homocysteine levels reducing: over the subsequent 2 years, almost all levels have been within the treatment target range (< 100 μmol/L).

### 4.4 Patient 5

Patient 5 was diagnosed with maple syrup urine disease during infancy after a metabolic decompensation following intercurrent illness. Shortly after diagnosis, concerns were raised regarding the family's ability to manage the patient during acute illness, including delays in contacting the on‐call team via phone, commencing the emergency dietary regimen, and attending the Emergency Department. This resulted in multiple episodes of severe illness (metabolic encephalopathy) due to delayed presentation. The patient had periods of weight loss without explanation and evolving developmental delay. These clinical events occurred despite regular attempts at intensive teaching sessions using different modes of communication (face‐to‐face with interpreter, telephone, written information, information in pictorial form).

The family was referred to the community social worker and a local psychosocial assistance service. This identified the parents' own learning difficulties and enabled respective support for them. The social worker regularly liaised with the child community nursing team to ensure that increased support was provided, including assessment by a child community nurse three times per week.

With further episodes of delayed response to acute illness, the level of concern was escalated to a child protection meeting. This led to social services increasing support to a comprehensive care package that enabled provision of a carer to prompt the family when feeds or medications were due, and also for when they needed to contact the on‐call metabolic service. With the family's capabilities improving, the care package was reduced after 12 months, and then ceased 6 months later, with leucine levels well‐controlled (< 400 μmol/L). The child's developmental trajectory normalized, albeit with persisting behavioral problems.

### 4.5 Patient 6

Patient 6 was diagnosed with a genetic hypertriglyceridemia during early infancy. The triglyceride level reduced to under 4 mmol/L with the institution of a medium chain triglyceride‐based formula as well as long‐chain fat restriction: it remained in the treatment target range of under 10 mmol/L for the next 6 months.

Over the next 2 years, triglyceride readings ranged up to approximately 40 mmol/L, with only one reading in the target range. This put the child at risk of acute pancreatitis [12]. This appeared to be due to non‐adherence to the prescribed very strict low‐fat diet, owing to challenges for the family (and family friends) in setting boundaries around food. Referrals to clinical psychology were made for behavioral intervention but were not attended by the family. Social care support was requested to facilitate a safer dietary environment; the patient was considered as a ‘child in need’ and support visits as well as “team around the family” meetings were undertaken.

It became apparent at these meetings that one of the main barriers to dietary compliance was parental limit‐setting, both at home and on the days when the patient spent time outside of the home. A management plan of increased parental supervision was created, with a plan to escalate to child protection meetings if unsuccessful. Following social care intervention, over the last 4 years, all except one of the patient's triglyceride level readings have been below 20 mmol/L, with the majority below 10 mmol/L. The patient continues to be well, with normal plasma lipase levels and no clinical episodes of pancreatitis.

## 5 Discussion

Non‐adherence to medical treatment is common in the pediatric population. Even in the Emergency Department setting, post‐discharge medication non‐compliance is reported to occur in over 25% of children [13]. In chronic diseases such as asthma, the risk of non‐adherence with daily medication is increased further [14].

Patients with many small molecule IEMs may similarly require daily medications [6], but also often require lifelong adherence to an extremely restrictive diet. Strict dietary adherence, especially in early childhood, results in much improved patient outcomes [15]. However, the intensive burden placed on families by these dietary prescriptions is underappreciated, with substantial impact on quality of life [16, 17]. This burden includes rigorously counting dietary macromolecules (including label reading as well as weighing of food), preparing appropriate meals, ensuring adequate caloric intake, recognizing illness early, and instituting appropriate management.

In IEMs that require long‐term dietary therapy, it is unsurprising, therefore, that biochemical evidence of sustained non‐adherence occurs in a significant minority of patients at some point during childhood [9, 18]. Similar to what has been reported in patients with Type 1 diabetes mellitus [19], adherence to diet is often considered more difficult than adherence to daily medication dosing. While it is established that medical practitioners often underestimate the degree of non‐adherence in their own patients [20], in the field of IEMs, biochemical monitoring often provides a specific way of measuring treatment adherence. This can then flag the need for interventions to support families to improve clinical and biochemical outcomes [21].

Despite the crucial role that these interventions often play in supporting dietary adherence in IEMs, there is relatively limited literature regarding the topic. A PubMed search (using key terms “inborn error of metabolism OR phenylketonuria” AND “diet adherence OR diet compliance” AND “social OR psychosocial OR child protection”) identified 104 results, of which 42 focused on dietary adherence in IEMs, with only two focused on impacts of social care involvement. Replacing the disease search terms with ‘diabetes’ resulted in an initial return of 705 articles, but again only six articles specifically focusing on social work interventions for patients with Type 1 diabetes mellitus. This suggests that for such diseases where dietary adherence is required to prevent adverse outcomes, there is very limited specific literature.

The risks posed by poor dietary control vary depending on the individual metabolic condition. In phenylketonuria (Cases 1–3), the risk primarily pertains to long‐term (developmental) outcomes [11]. In conditions that can present more acutely (Cases 4–6), there is also a superimposed risk of metabolic decompensation (vascular events in CBS deficiency, potentially lethal metabolic encephalopathy in MSUD, acute pancreatitis in genetic hypertriglyceridemias) [12, 22, 23].

**FIGURE 2:**
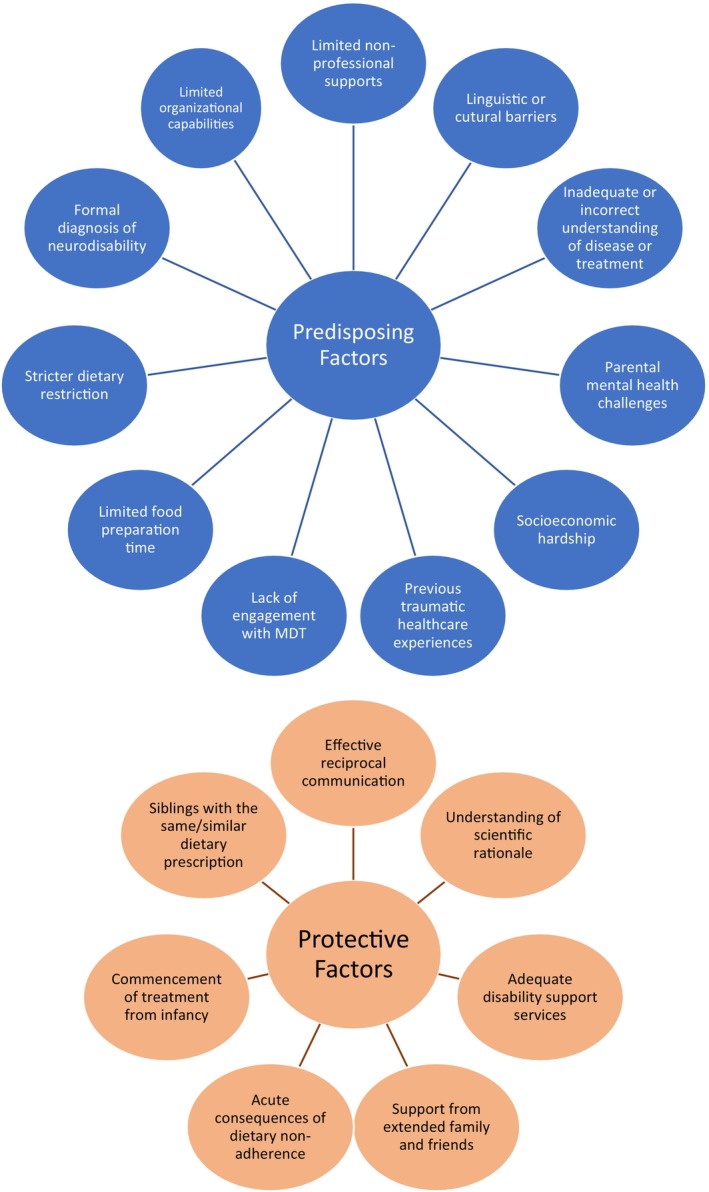
Predisposing and protective factors influencing dietary non‐adherence in patients with inborn errors of metabolism.

Families manage this substantial burden with varying degrees of success, impacted by a number of “predisposing factors” and “protective factors”: those pertinent to IEMs have been extracted from the available pediatric adherence science literature, and summarized in Figure 2 [4, 8, 24, 25, 26, 27, 28, 29]. Families facing multiple “predisposing factors” with a paucity of “protective factors” may require more support to facilitate treatment. It is important that healthcare practitioners recognize families where this imbalance exists and begin by devising a treatment plan from the outset that is pragmatic. Acknowledging differing health beliefs and the difficulties of adherence, highlighting availability of (and using) interpreters when required, and compromising where possible are also crucial [30, 31].

Once these recommendations have been decided on, there are many strategies to support adherence to treatment, some of which were outlined by van Rijn and colleagues [21]. Many of these, such as providing an education package, exploring alternative treatment options, and hospital admission to demonstrate that biochemical control can be achieved, are able to be facilitated by the metabolic team itself. However, others, particularly recruiting community supports, psychometric or mental health assessments of other family members, and involvement of child protection services, often require liaison with other organizations and can be facilitated by liaison with social care.

Across our cases, there were some common themes identified that suggested the need for social care involvement. Failure of the family to engage with the multidisciplinary team was evident, often across different disciplines (medical, dietetics, nursing) and across modes of communication (in‐person reviews, phone calls, written communication). Some patients also had clinical trajectories that were out of keeping with their initial disease manifestations. For instance, Patient 5 had presentations that were unusually severe for a patient with an intermediate form of MSUD. In all cases, the burden of the diet challenged the family's ability to manage, resulting in sustained or excessive biochemical derangements.

The role of social care in improving clinical outcomes varied across our cases. For Patient 1, a social worker assisted in solving logistical problems (such as delays in arrival of specialized feeds) and helped expedite housing repairs. For Patients 2 and 3, referral to social care mandated regular dried blood spot monitoring, encouraged parents to seek support for their mental health, and eventually resulted in the assumption of care. In Patient 4, where concerns were more acute, the involvement of a social worker facilitated rapid escalation to child protection services, resulting in greater family engagement. For Patient 5, a case conference led by social work resulted in the institution of a 24‐h carer package to support parents with their own cognitive difficulties. For Patient 6, referring the family to social care helped emphasize the gravity of the situation and resulted in improved (and sustained) dietary adherence. This demonstrates the varied and gradually escalating ways in which social care can support the metabolic multidisciplinary team and is summarized in Figure 1.

Another important consideration underscoring the need for social work involvement is that patients with IEMs are often picked up on newborn screening. A key tenet of screening, even in the original principles set out by Wilson and Jungner, is that ‘facilities for diagnosis and treatment should be available’ [32]. The facilities for treatment includes not only medical care, but also psychological, social and economic supports for families [33]. Having access to a social worker as part of the clinical team would help to identify socioeconomic issues from the outset and likely prevent escalation at a later stage.

We have experienced recurrent challenges when working with local social work teams. The large catchment area of our service and localized social work teams with high staff turnover mean that each individual social worker requires education by the metabolic team regarding the specific child's medical condition and the required management to prevent harm. In addition, from a child protection perspective, two key points need to be conveyed. First, that the threshold for seeking clinical review (either via telephone or in the Emergency Department) should be lower in a child with an IEM; and secondly, ensuring understanding that for disorders without acute toxicity (e.g., phenylketonuria), developmental and other medical consequences of dietary non‐adherence are likely to be insidious [11]. Under such circumstances, limited understanding of social workers of the risk of undertreatment in disorders with insidious onset of harm can lead to rejection of referrals or early discharge from social care in the absence of other indicators of neglect.

Other challenges faced by local social work teams when managing metabolic patients include a lack of protected time for metabolic case management and misunderstandings created by indirect methods of communication. A social worker external to the metabolic department may also be perceived as more intimidating by families (seeing them as part of a child protection framework), rather than a specific metabolic social worker who is involved from the outset in helping families adjust to a new diagnosis. Having an on‐site metabolic social worker, liaising with local practitioners including health visitors and community nursing, would be beneficial in minimizing these issues. Despite this, in a survey of over 900 patients or caregivers of patients with IEMs, only 28% had guaranteed access to a social worker [34]. This demonstrates a clear area of unmet need in the holistic management of these patients.

### 5.1 Limitations

As with any intervention, there are instances where social work involvement does not necessarily result in improvement in biochemical control. We had a further 12 such patients, including some with PKU, MSUD, homocystinuria, and other rare metabolic disorders. In these cases, the main factor that limited the intervention was the lack of engagement of patients or parents with social care, often in the context of additional psychosocial stressors such as homelessness, parental childhood trauma, or intimate partner violence. In many of these families, dietary management of the metabolic disorder was understandably not the primary focus. Unsuccessful interventions were also often associated with a delayed referral to social care, after prolonged periods of non‐adherence, especially in older children. Trying to avoid a potential conflict with the family and a perception of futility may have prevented the clinical team from making an early referral in some cases. We believe that outcomes for many of these families could be improved nonetheless if a social worker who is part of the metabolic team can build rapport with a family right from the time of diagnosis, facilitating much earlier intervention.

This paper details the perspective of the metabolic team regarding the importance of integrated social care. We acknowledge that the family perspective should also be considered and evaluated. Social care referrals took place after discussion with families and with their consent. While the metabolic team performs regular patient satisfaction surveys, we do not generally ask for feedback about the provision of psychosocial support (such as assisting with adjustment to acute illness, addressing psychological barriers to dietary adherence) or about specific social work services (e.g., ensuring access to adequate education, housing and relevant financial support) as this only concerns a small proportion of our patients. The effectiveness of social care interventions could be assessed by comparing centers with and without integrated social care involvement, or at a single center following the introduction of integrated social care. In teams where a social worker is integrated, longitudinal studies could help estimate the frequency of patients with IEMs requiring social care support, to ensure adequate staffing across centers.

## 6 Conclusion

Small molecule inborn errors of metabolism require complex and intensive dietary management, which places a substantial burden on families. In families where the psychosocial and medical challenges overwhelm their coping strategies and external supports, treatment adherence becomes difficult. This in turn may lead to either sustained biochemical derangement or acute decompensations, both of which can result in suboptimal patient outcomes. In this paper, we have presented case vignettes demonstrating the positive role that social care involvement can play, in addition to intensive psychosocial support by a multidisciplinary clinical team. We want to encourage clinicians to consider a referral to social care at an earlier stage and to advocate for the inclusion of a social worker in the clinical team: to build trust with the family from the time of diagnosis, enhance knowledge sharing among healthcare professionals and social services, and to provide a more holistic experience for families.

## Author Contributions

A.S.: data collection, writing of manuscript, creation of figures, drafting of manuscript, final proofreading. S.N.: creation of figures, drafting of manuscript, final proofreading. K.v.W.: drafting of manuscript, final proofreading. E.S.: drafting of manuscript, final proofreading. V.H.: drafting of manuscript, final proofreading. R.H.: drafting of manuscript, final proofreading. F.W.: drafting of manuscript, final proofreading. B.C.S.: study conceptualization, writing of manuscript, drafting of manuscript, final proofreading.

## Ethics Statement

No ethics approval was sought for this study: instead every effort was made to anonymize the case vignettes.

## Conflicts of Interest

The authors declare no conflicts of interest.

## Data Availability

Access to de‐identifed patient data will be made available from the corresponding author on reasonable request.
